# The Influencing Factors of Nutrition and Diet Health Knowledge Dissemination Using the WeChat Official Account in Health Promotion

**DOI:** 10.3389/fpubh.2021.775729

**Published:** 2021-11-25

**Authors:** Dongsheng Bian, Yongmei Shi, Wenjia Tang, Dong Li, Kangni Han, Chenshu Shi, Guohong Li, Fan Zhu

**Affiliations:** ^1^Department of Clinical Nutrition, Ruijin Hospital, Shanghai Jiao Tong University School of Medicine, Shanghai, China; ^2^School of Public Health, Shanghai Jiao Tong University School of Medicine, Shanghai, China; ^3^Department of Hospital Publicity, Ruijin Hospital, Shanghai Jiao Tong University School of Medicine, Shanghai, China; ^4^Center for Health Technology Assessment, China Hospital Development Institute, Shanghai Jiao Tong University, Shanghai, China

**Keywords:** WeChat, nutrition and diet, health promotion, Healthy China, social media

## Abstract

**Background:** The promotion of a healthy diet *via* health education is a component of the “Healthy China 2030” plan. However, few studies have reported whether health knowledge about nutrition and diet has gained public attention, and whether it is needed by the public.

**Methods:** The numbers of views, shares, and reads of articles published by the official WeChat account of a hospital in China were accessed. The influence index was obtained *via* the entropy analysis of these three indices. A questionnaire survey was developed based on the purpose of the study and the conclusion of the content analysis, which conducted to analyze users' requirements for health knowledge and their influencing factors. Moreover, risk factors were explored by logistic regression models.

**Results:** Of the 103 articles considered in this study, four articles in the Top 10 were related to nutrition and diet. The influence index of nutrition and diet knowledge was found to be the highest in the content analysis (*p* < 0.05). The higher degrees of humor (β = 0.224, *p* = 0.027), nutrition and diet articles (β = 0.776, *p* = 0.034), and cover articles (β = 0.312, *p* = 0.021) have significant influences on the influence index. In total, 581 questionnaires were obtained, and 78.1% of the respondents reported believing that the health knowledge of greatest concern was that related to nutrition and diet. Multivariate logistic regression analyses were conducted to explore the associations between the features of the articles and users reading nutrition and diet knowledge; it was found that gender (female, OR: 4.651, 95%Cl: 2.598, 8.325, and *p* < 0.001), age (young adult, OR: 0.358, 95%Cl: 0.266, 0.481, and *p* < 0.001), cancer precaution knowledge (OR: 4.333, 95%Cl: 2.262, 8.299, and *p* < 0.001), traditional Chinese medicine (OR: 2.121, 95%Cl: 1.064, 4.230, and *p* = 0.033), the knowledge acquisition approach [circle of friends (OR: 2.586, 95%Cl: 1.373, 4.868, and *p* = 0.003), social media (OR: 2.183, 95%Cl: 1.204, 3.960, and *p* = 0.010)), hospitals (OR: 3.194, 95%Cl: 1.793, 5.692, and *p* < 0.001), television media (OR: 4.348, 95%Cl: 2.341, 8.077, and *p* < 0.001)], and social media strategies [professionalism and authority (OR: 2.354, 95%Cl: 1.231, 4.505, and *p* = 0.006)] have statistically significant relationships with users reading nutrition and diet knowledge.

**Conclusion:** Nutrition and diet knowledge could contribute to WeChat user engagement of health information dissemination. Nutrition professionals should improve the scientific popularization ability and effectively use social media for health promotion.

## Introduction

Due to the growing prevalence of diet-related noncommunicable diseases, especially those exacerbated by rising rates of obesity and improvements in survival, the total number of obese adults in China reached 85 million in 2018 (1). In 2016, the number of deaths of Chinese people related to diet-related chronic diseases reached 2.493 million, accounting for 29.1% of all deaths from chronic diseases in the country, with a mortality of 182.4/100,000 (2). Studies have shown that an unhealthy diet, is the main behavioral risk factor for the development and progression of chronic diseases (3). Evidence based on macro-data from 1961 to 2017 revealed that the structural imbalances in dietary consumption in China have seriously threatened national health (4). In the past 30 years, the diet of Chinese people has changed from the traditional plant-based diet to a Western-style diet, which is mainly reflected in the increased intake of animal products and the consumption of fine processed grains, sugary drinks, and highly processed foods (5). Another key factor is that increasingly more young and middle-aged people use take-out services, which increases their consumption of foods high in sugar, salt, fat, and calories (6). Because these unhealthy diet behaviors affect health, it is necessary to strengthen health education and guide healthy dietary behavior. “Healthy China” is an important strategic policy with obvious Chinese characteristics implemented by the Chinese government, and it has been deemed important to change the living and dietary habits of Chinese people *via* health education (7).

The acquisition and dissemination of health information play a significant role in promoting positive behavioral changes related to health (8), and social media is currently becoming a new channel for information acquisition and exchange (9). With the rapid development of new media, mobile phone-centered intelligent devices are gradually covering more areas of social life and are a popular means of information acquisition and exchange by the public (10). Network platforms are the major distribution centers of information flow. Furthermore, the demand for health information is also surging, and the use of social media to obtain health information has gradually become the norm (11, 12). Recent evidence suggests that social media platforms, such as Facebook and Twitter, are being used to promote health management and education, increase doctor-patient communication, and provide direct services (13, 14).

Many internationally popular social media apps, such as Twitter, Facebook, and Instagram. WeChat is a mobile social app launched by Tencent in 2011, and supports the functions of sending text, voice, and video messages, multi-person voice intercom, and reading and sharing information. As of January 2021, the app, which is China's most active social media platform with 1.225 billion monthly active users, has had an undoubtedly substantial impact on the communication and lifestyle of users (15). The widespread public engagement *via* WeChat creates a ready platform for successful online information distribution and diffusion by health promotion agencies (16). During the outbreak of the COVID-19 pandemic, WeChat official accounts (WOAs) provided necessary medical support for the public, reduced social panic, promoted social isolation, enhanced the self-protection ability of the public, and promoted epidemiological screening, thereby playing an important role in preventing and controlling the spread of COVID-19 (17).

Communication and education are inextricably linked to the effective dissemination and uptake of health information. Previous studies on online health communication largely focused on the influencing factors of user engagement with disseminated health information, including highlighting celebrity involvement, the use of humorous appeals, etc. (18, 19). Zhang et al. (20) reported that article content, article type, communication skills and article length were associated with user engagement. Gabarron et al. (21) revealed that health education on social media, such as Facebook and Instagram should consider videos and emoji in their posts to increase user engagement. Jenkins et al. (22) found that nutrition professionals should convey positive emotions and success to enhance the trustworthiness of their posts. However, there remains a lack of quantitative analysis and empirical research on how to accurately parse out the health needs of users from massive amounts of health information.

According to use and gratification theory, users actively select the type and content of information to satisfy their needs, and media that provide the most satisfying content will be used more often than others. Thus, it is necessary to evaluate and understand users' cognition of and attitudes toward the contents of media. WOAs edit and publish articles, and then push them to their followers, who spread the articles by reading and sharing them with their circle of friends. Thus, the numbers of views, reads, and shares have become important criteria by which to judge the attention paid to articles. Professional medical institutions are the main bodies of health information dissemination in China.

Public hospitals, especially top tertiary hospitals, have the most adequate medical personnel, resources, and technology, and are the main providers of health information. Shanghai Ruijin Hospital is one of top tertiary hospitals in China. As the affiliated hospital of Shanghai Jiao Tong University, Ruijin hospital is a leading general hospital incorporating research, education and care. Based on the heritage of Western medicine and enriched by Chinese medicine and culture, the hospital has established a strong global reputation for medical innovation. Besides, the WOA of Shanghai Ruijin Hospital is also a benchmark in the field of health promotion. The influence of Shanghai Ruijin Hospital's WOA ranks among the top 20 hospitals in China.

The promotion of a healthy diet *via* health promotion and education is an important component of the “Healthy China 2030” plan. However, few studies have reported whether health information about nutrition and diet has gained public attention, and whether it is needed by the public. The research questions in this paper are as follows:

1. Whether health information about nutrition and diet has gained public attention, and whether it is needed by the public?
2. What were the influencing factors and the key elements of nutrition and diet health knowledge dissemination?
3. What techniques are associated with greater user engagement with nutrition and diet health knowledge?

In the present study, the WOA of Shanghai Ruijin Hospital was surveyed. By describing the numbers of views, shares, and reads of WeChat articles, the types of health knowledge most needed by users were determined, and factors that promote the wide dissemination of health communication were acquired. Then, a questionnaire was designed based on the content analysis to provide evidence to evaluate the effectiveness and practicality of using WOAs in public health education.

## Materials and Methods

### Content Analysis

The WOA of Shanghai Ruijin Hospital (https://mp.weixin.qq.com/mp/profile_ext?action=home&__biz=MzIwNzEwOTM2MA==&scene=124) was chosen as the study subject. Beginning on April 17, 2020, it officially published two to four health knowledge articles every week. As of June 11, 2021, a total of 101 health knowledge articles had been published. These articles were used as samples for quantitative analysis in the present study to better understand users' preferences and make early preparations for the questionnaire surveys conducted in the later stage of research.

### Data Collection

The data used in this study were the backstage data of the WOA, including the numbers of articles published, viewed, shared, and read. At the bottom of each article, the options “like” and “reading” can be selected. The selection of “like” indicates the recognition of the article, which is then seen by a user's followers. The selection of “reading” allows the users' friends to see the article, interact with each other, and carry out secondary dissemination (23, 24). Moreover, a six-category feature framework by which to consider each article was developed, referring to the research of Kite et al. (18) and Zhang et al. (20), with modification made during iterative testing to ensure consistency across features and make feature framework more relevant to effectiveness of the WOA. Specifically, we added three degrees of humor was adapted from the research of Kuangand Wu (25), which is feature that may affect the health communication in WOA. Such as, “*I am a mosquito and I prefer to bite fat boys, ahahaha…*” and the whole article was cheerful, we defined this article as high degree of humor. “*Elevated tumor markers* = *tumor? You must promptly seek medical advice if you have these eight big signs*” and nearly half of the article is humor written, we defined this article as normal degree of humor. “*Muscle soreness is a side effect of the COVID-19 vaccine*” and the description of the whole article is very rigorous, we defined this article as low degree of humor. Coding of all feature framework was completed independently by all authors, with any discrepancies resolved through discussion. The final feature framework and related definitions are reported in Table 1.

**Table 1:** The final feature framework used in this study.

**Item**	**Definition**
**Article content**	
Nutrition and diet	The topic is related to nutrition or diet, e.g., the topic introduces the effects of food and nutritional therapy
Covid-19	The topic is related to COVID-19, e.g., the topic describes vaccines and precautions.
Healthy lifestyle	The topic is related to popular knowledge of life, e.g., ways to protect your eyes, the dangers of sedentariness, etc.
Chronic diseases	The topic is related to chronic diseases, e.g., the prevention and therapy of non-alcoholic fatty liver disease, etc.
Clinical trials	The topic is related to clinical trials, such as those for Parkinson's disease.
Digital healthcare	The topic is related to digital health, such as how to see a doctor online.
Cancer precaution	The topic is related to cancer, such as tumor markers.
**Degree of humor**	
Low	No humorous technique is used to convey health messages.
Normal	A slightly humorous technique (such as slight sarcasm, jokes, etc.) is used to convey health messages.
High	A very humorous technique (such as overt sarcastic, jokes, etc.) is used to convey health messages.
**Presence of caricatures**	
Text only	The article contains only text.
Text and caricatures	The article contains text and caricatures.
**Article length**	
0–1,500 words	The number of words in the article text.
1,500 or more words	The number of words in the article text.
**Title type**	
Declarative sentence	The sentence aims to state a fact with a period.
Exclamatory sentence	The sentence aims to state a fact with an exclamation point.
Interrogative sentence	The sentence has a question mark.
Imperative sentence	The sentence has an exclamation point without a subject.
Cover article	Whether the article is a cover article.

### Questionnaire Survey and Quality Control

A questionnaire was developed based on the purpose of the study and the conclusion of the content analysis, and primarily included questions on each respondent's age, gender, educational background, occupation, accessibility and frequency of access to health information channels, types of health knowledge needs, and social media strategies. Respondents' attitudes toward each statement were measured on a 5-point Likert scale (1 = strongly disagree, 5 = strongly agree). When the preliminary design of the questionnaire was completed, it was reviewed by experts familiar with the Research Topic, who ensured that the survey questions successfully captured the topic and did not contain common errors such as leading, confusing, or double-barreled questions. Prior to the survey, a pilot study was conducted among 20 subjects to ensure that there were no problems in reading the frame information, understanding and answering the questions in the questionnaire. All participants said the frames were easy to understand and the length of the questionnaire was appropriate.

Quality control measures: the questionnaire was accompanied by detailed instructions and informed consent. All survey respondents must follow and read more than five health articles in the WOA of Shanghai Ruijin Hospital. Besides, we set all the questions must be completed before submitted the questionnaire. Additionally, we set the same IP or WeChat account can only be filled once to prevent repeated filling. We also set a limit on the time required to fill the questionnaire. All questionnaires that taken < 1 min to answer were excluded. Moreover, the same answers for consecutive questions also were excluded. Finally, a total of 21 questionnaires were excluded, resulting in 581 valid questionnaires.

The questionnaire survey was conducted on June 25, 2021 *via* the function of “reading” the original article at the bottom of the WOA. The data used in this study were sourced from the backstage management function of the WOA and stored in the cloud network. The questionnaire survey was also conducted online, which ensured reliable data sources and accurate content.

Principal component analysis (PCA) was then performed, and the questionnaire was checked for internal consistency. According to the results of PCA and internal consistency, several disturbance terms were deleted. The Kaiser–Meyer–Olkin measure of sampling adequacy was 0.783, the *p*-value of the Bartlett test of sphericity was < 0.001, and Cronbach's alpha was 0.763.

### Calculation Formula of Influence Index and Statistical Analysis

Hirsch (26) proposed an index *h*, defined as the number of papers with citation number ≥h, as a useful index to characterize the scientific output of a researcher. A high *h* is a reliable indicator of high quality of the researcher's research paper and high accomplishment. WOA is equivalent to a researcher, the articles published by WOA were similar to the research paper published by researchers. The number of views and reading of articles published by WOA also had similar characteristics to those cited by researchers. The WeChat communication index (25) was established the dissemination and coverage of messages published by WOA as well as the maturity and impact of WOA. In our research, the influence index of articles was derived from rigorous calculation formulas adapted from WeChat communication index (27), Kaur (23), and Hirsch (26), which consist of three dimensions, that is, *viewing, reading*, and *sharing*.

Given that the number of people each article was sent to was different, and because the sending situation has a great impact on reading, the number of views was transformed by the number of serviced people; the viewing index = 100 × number of views/number of sends. Moreover, because sharing and collection occur on the premise of reading, and represent the readers' recognition of the article, the number of views was used to standardize the number of shares and reads; the sharing index = 100 × number of shares/number of views, and the reading index = 100 × number of reads/number of views. The *z*-scores for the viewing index, sharing index, and reading index were computed to create a standardized score for each.

After standardization, descriptive analysis was performed on the *z*-scores of the viewing, sharing, and reading indexes. For each domain, we incorporated the values of the *z*-scores of the viewing, sharing, and reading indexes into the scoring by using entropy weight, in which weights are systematically calculated based on the level of the difference between the original values (28).

Entropy weight method:

Matrix after logical transformation:


(1) $$\begin{array}{r} M=\left(\begin{array}{ccc} x1 & \cdots & x1m\\ \vdots & \ddots & \vdots \\ xn1 & \cdots & xnm \end{array}\right) \end{array}$$

Where n: number of health articles; *m*, number of variables.


(2) $$\begin{array}{r} P_{ij}\;P_{ij}=\frac{x_{ij}}{\sum_{i=1}^{n}x_{ij}} \end{array}$$

(3) $$\begin{array}{r} E_{j}\;E_{j}=-\frac{1}{\ln\left(n\right)}\sum_{i=1}^{n}P_{ij}\ln\left(P_{ij}\right) \end{array}$$

(4) $$\begin{array}{r} d_{j}\;d_{j}=1-E_{j} \end{array}$$

(5) $$\begin{array}{r} W_{j\left(weight\;for\;each\;z-indexes\;\right)}\;W_{j}=\frac{d_{j}}{\sum_{j=1}^{m}d_{j}} \end{array}$$

Collinearity diagnostics were determined *via* the tolerance statistic and variance inflation factor (VIF), which revealed that collinearity was not a problem between covariates (29). The differences in the z-scores of the viewing, sharing, and reading indexes and the influences of nutrition and diet-related articles and other types of articles were studied by a univariate analysis of variance. Next, stepwise regression analyses were conducted to assess the associations between the features of the articles and the influence index (described in section Analysis of Article Influence).

Microsoft Excel software was used to create a database for data entry and verification, and data were cleaned after entry to form the database. SPSS 22.0 software was employed for data analysis.

The odds ratio (OR) and its 95% confidence interval (CI) were calculated to determine the association of risk factors with nutrition and diet knowledge. Variables with *p* < 0.5 in the univariate analyses were then included in a multivariate regression model to identify the independent risk factors *via* backward elimination analysis. The variables included in the multivariable logistic regression analysis were included as categorized variables. A test level of α = 0.05 and *p* < 0.05 was considered statistically significant.

## Results

### Basic Information

On April 27, 2020, the number of followers of the Shanghai Ruijin Hospital WOA was 661,223. By June 11, 2021, the number was 1,120,533, of which female followers accounted for 59.73% (669,350) and male followers accounted for 40.21% (450,624). Moreover, 69.19% (767,061), 10.74% (119,033), and 7.51% (83,256) of the followers were from Shanghai, Jiangsu Province, and Zhejiang Province, respectively.

### Basic Information of Articles

**Figure 1:**
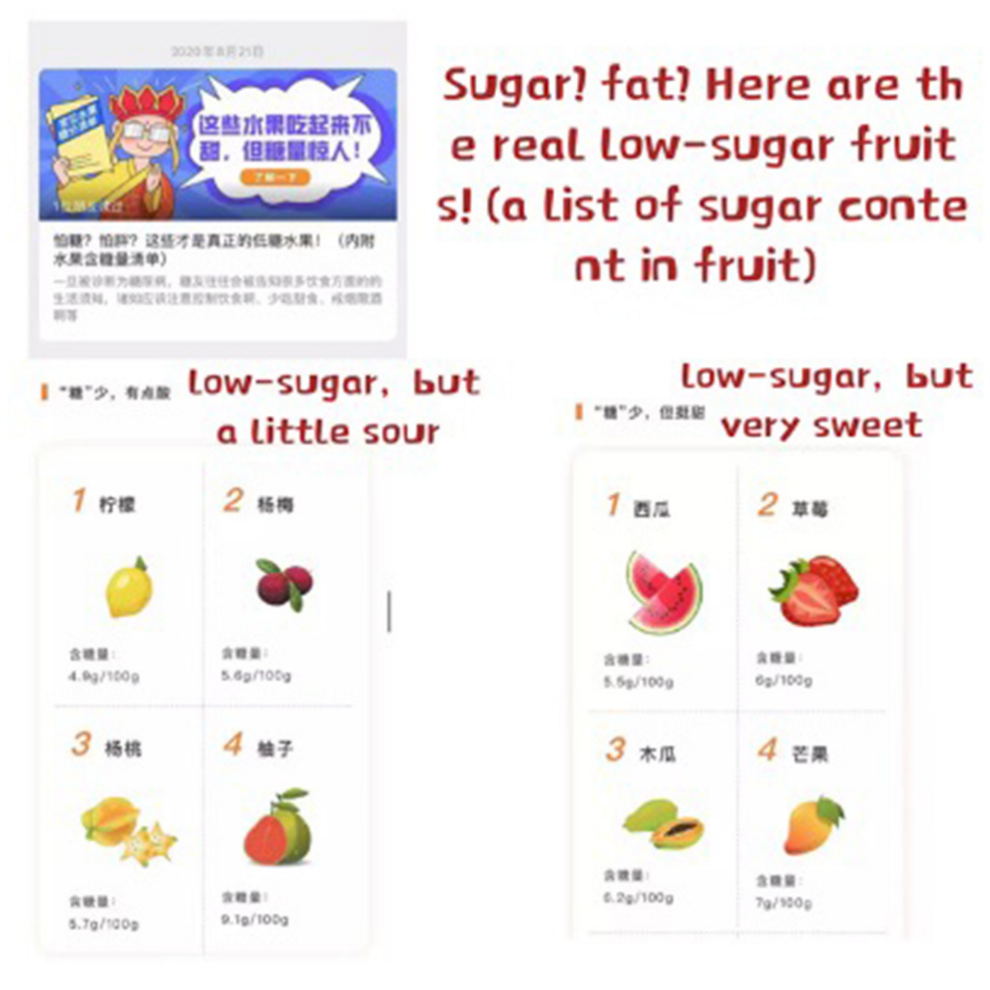
A screenshot of a counter-rumor article. Source: Shanghai Ruijin Hospital WOA; online: https://mp.weixin.qq.com/s/Ltizmq1x6plwvSacAjjbYw (accessed August 21, 2020).

The total number of views of health knowledge articles was 4,832,682, the maximum number of views of one article was 326,274, the average number of views was 48,814, and the minimum number of reads was 1,809. Due to the settings of the Tencent system, a number of views of more than 100,000 is expressed as “100,000+,” which is considered the most popular. Thirteen of the 103 articles considered in this study had more than 100,000 views, four of which were on nutrition and diet (Table 2, Figure 1).

**Table 2:** Health knowledge articles with 100,000+ views.

**Title (English)**	**Views**
Sugar? Fat? Here are the real low-sugar fruits! (A list of the sugar contents of fruits)	326,274
What should your daily diet be if your thyroid gland has a problem?	219,859
Elevated tumor markers = tumor? You must promptly seek medical advice if you have these eight big signs	143,046
How to take a good nap!	127,830
Look here! “Fast track” of Shanghai Ruijin Hospital COVID-19 nucleic acid detection	126,752
Skin disease, hypertension, thyroid disease? If you have several types of chronic diseases, can you get the COVID-19 vaccine?	123,668
Diego Maradona died of a heart attack. Five things you need to know to save your life	123,619
What is the “antibody cocktail” that Donald Trump is receiving?	119,394
Why are your eyelids twitching all the time?	118,607
Did the use of a humidifier like this cause 14,000 deaths? A doctor says that these four points should be considered!	115,682
Eating like this every day can improve the body's immunity	107,598
Where is the “held fart”? Will it affect your health?	102,294
What is the effect of red bean Semen Coicis water on the human body?	101,575

### Analysis of Article Influence

To comprehensively present the numbers of views, shares, and reads of the article, the entropy method was used to determine weights, and the influence index was used to comprehensively describe the influence levels of the articles.

**Figure 2:**
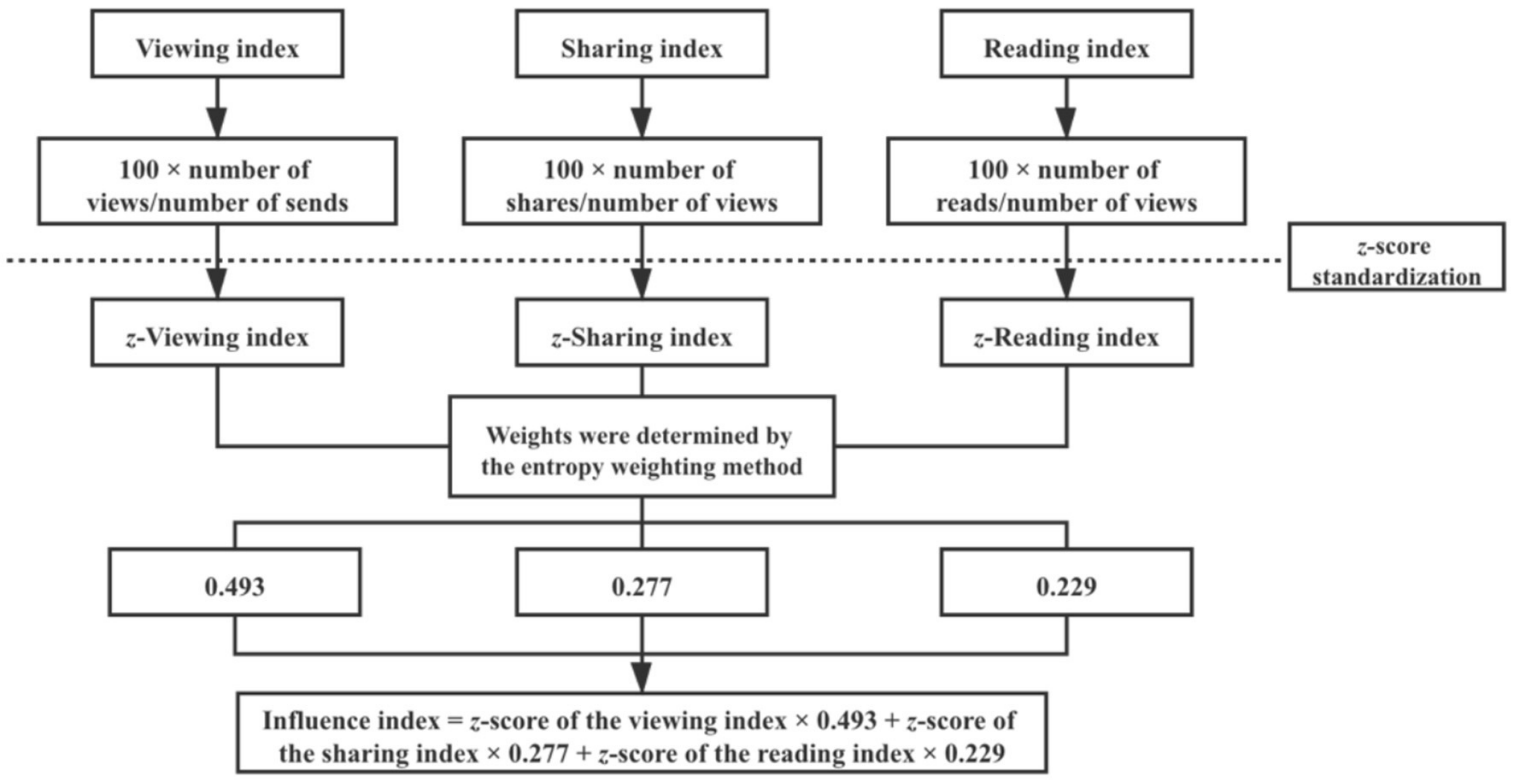
Steps of influence index calculation.

The weights of the viewing index, sharing index, and reading index were, respectively, 0.493, 0.277, and 0.229. According to the results of the entropy method analysis, the influence index = *z*-score of the viewing index × 0.493 + *z*-score of the sharing index × 0.277 + *z*-score of the reading index ×0.229 (Table 3, Figure 2).

**Table 3:** The analysis of the influences of different article contents (median).

**Category of health knowledge**	**Accumulated articles**	**Viewing index**	**Sharing index**	**Reading index**	**Influence index**
Nutrition and diet	22	0.166	0.405	0.204	0.367
Healthy lifestyle	26	−0.241	−0.131	−0.412	−0.308
COVID-19	12	−0.321	−0.101	−0.049	0.186
Chronic disease	21	−0.598	0.220	−0.045	−0.378
Digital healthcare	14	−0.334	−0.592	−0.430	−0.209
Clinical trials	3	−0.769	−0.355	−0.131	−0.6215
Cancer precaution	5	−0.020	−0.168	−0.626	−0.070
*K*		14.316	11.045	6.784	14.102
*P*		0.026	0.087	0.341	0.029

A rank-sum test was performed, and the results revealed significant differences between the viewing index and influence index of articles with different topic categories (*p* < 0.001).

### Multiple Regression Analysis of the Influence Index of Health Knowledge

Table 4 reports the multiple linear regression results based on the least-squares method and stepwise regression. The results reveal that higher degrees of humor (β = 0.224, *p* = 0.027), nutrition and diet health articles (β = 0.776, *p* = 0.034), and cover articles (β = 0.312, *p* = 0.021) have significant influences on the influence index.

**Table 4:** The stepwise regression analysis of the influence index of health knowledge.

	**β**	***t***	***p***
Degree of humor	0.224	2.246	0.027
Nutrition and diet health articles	0.776	2.148	0.034
Cover article	0.312	2.352	0.021

### Demand for Nutrition and Diet Health Knowledge by Questionnaires

**Figure 3:**
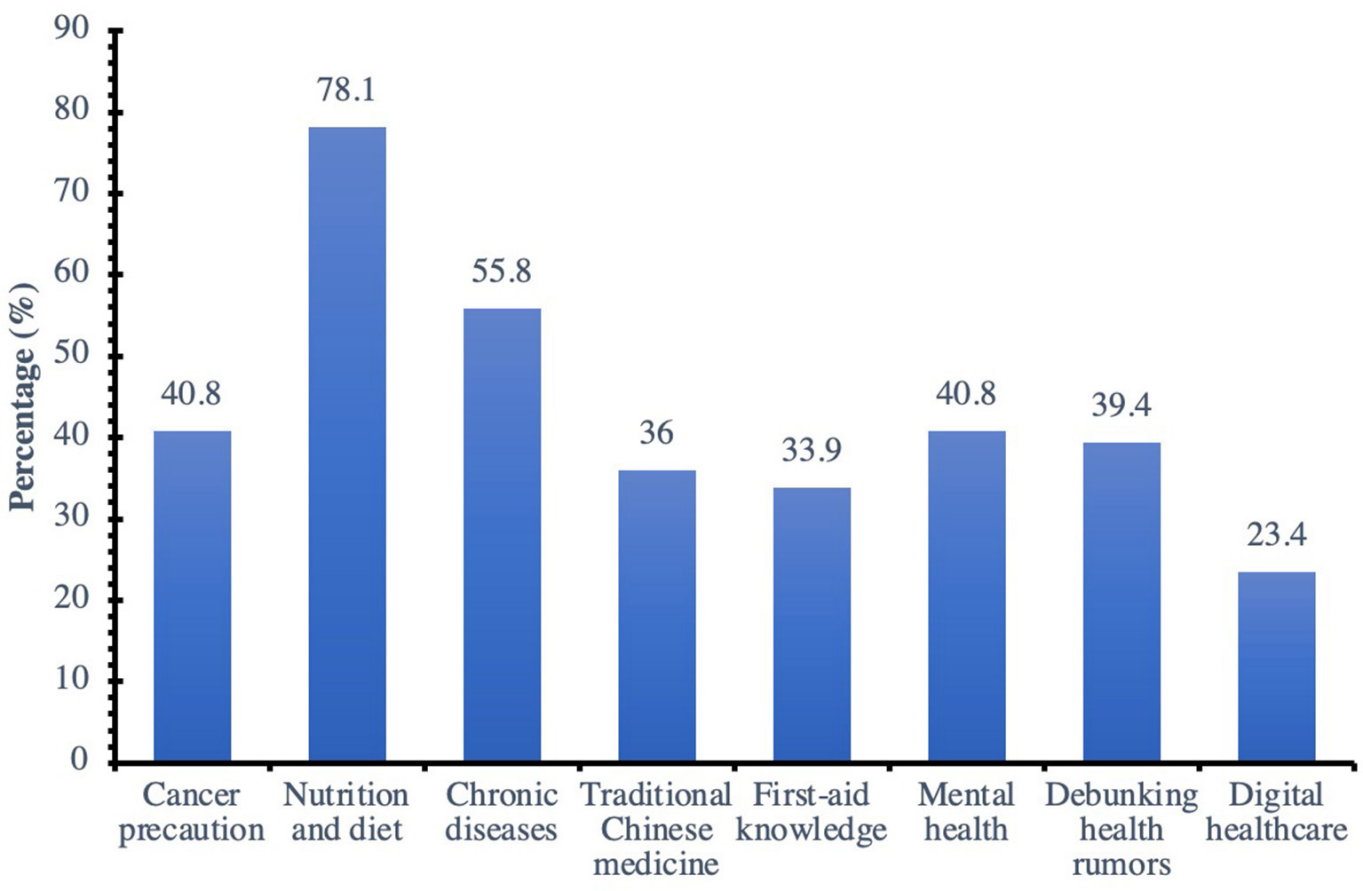
What type of health knowledge are you most concerned?

Feedback was collected from a total of 581 users, 196 of whom were male respondents. Of the users, 91.9% took the initiative to acquire health knowledge, 75.9% reported that WOAs and other social media were major sources of health information, and 78.1% reported that nutrition and diet information is the health knowledge of greatest concern. In addition, 69.38% (403), 10.00% (58), and 3.10% (18) of the followers were from Shanghai, Jiangsu Province, and Zhejiang Province, respectively. The population distribution of respondents was basically consistent with the distribution of WOA of Shanghai Ruijin Hospital followers (Table 5, Figure 3).

**Table 5:** Respondents' demand for health knowledge.

**Basic information**	**Number of cases**	**Percentage (%)**
**Gender**		
Male	196	33.7
Female	385	66.3
**Age distribution**		
≤ 35 years	213	36.7
35–45 years	112	19.3
45–60 years	96	16.5
≥60 years	160	27.5
**Whether you actively acquire health knowledge**		
Yes	534	91.9
No	47	8.1
**Where you get your health knowledge**		
Newspapers and books	249	42.9
Television media	283	48.7
Internet search	293	50.4
WOAs and other social media	441	75.9
Circle of friends	239	40.8
Hospitals	309	53.2
Off-line classes	185	31.8
**What type of health knowledge are you most concerned about?**		
Cancer precaution	237	40.8
Nutrition and diet	454	78.1
Chronic diseases	324	55.8
Traditional Chinese medicine	209	36.0
First-aid knowledge	197	33.9
Mental health	237	40.8
Debunking health rumors	229	39.4
Digital healthcare	136	23.4

### The Influencing Factors of Users Reading Health Articles About Nutrition and Diet Knowledge

The univariate analysis results showed that gender, age, the knowledge acquisition approach (circle of friends, WOAs and other social media, hospitals, off-line classes, television media and internet search), the contents of health knowledge (cancer precaution, traditional Chinese medicine, debunking health rumors, first-aid knowledge, and digital healthcare), and social media strategies (providing new health knowledge, being an important source of health knowledge, professionalism and authority, close-to-life articles, the use of caricatures, engagement with the public) are closely related to users reading articles of nutrition and diet knowledge (*p* < 0.5) (Table 6).

**Table 6:** The influencing factors of users' reading of nutrition and diet knowledge articles.

	style="border-bottom: thin solid #000000;"**Univariate logistic analysis**		style="border-bottom: thin solid #000000;"**Multivariate logistic analysis**	
	**OR (95% Cl)**	***p***	**OR (95%Cl)**	***p***
**Basic information**				
Gender	2.813 (1.879, 4.212)	0.000^*^	4.651 (2.598, 8.325)	0.000^*^
Age	0.538 (0.451, 0.641)	0.000^*^	0.358 (0.266, 0.481)	0.000^*^
**Knowledge acquisition approach**				
Circle of friends	2.572 (1.648, 4.014)	0.000^*^	2.586 (1.373, 4.868)	0.003^*^
WOAs and other social media	3.640 (2.384, 5.556)	0.000^*^	2.183 (1.204, 3.960)	0.010^*^
Hospitals	3.311 (2.173, 5.044)	0.000^*^	3.194 (1.793, 5.692)	0.000^*^
Off-line classes	1.676 (1.065, 2.637)	0.025^*^	0.916 (0.473, 1.772)	0.795
Television media	1.766 (1.180, 2.643)	0.006^*^	4.348 (2.341, 8.077)	0.000^*^
Internet search	1.385 (0.932, 2.057)	0.107^*^	0.718 (0.386, 1.337)	0.296
**Contents of health knowledge**				
Cancer precaution	3.793 (2.345, 6.136)	0.000^*^	4.333 (2.262, 8.299)	0.000^*^
Chronic diseases	1.120 (0.794, 1.579)	0.519		
Traditional Chinese medicine	2.952 (1.823, 4.780)	0.000^*^	2.121 (1.064, 4.230)	0.033^*^
First-aid knowledge	1.262 (0.825, 1.933)	0.284^*^	0.675(0.371, 1.226)	0.196
Mental health	1.034 (0.693, 1.545)	0.869		
Debunking health rumors	1.783 (1.165, 2.728)	0.008^*^	0.985 (0.510, 1.901)	0.964
Digital healthcare	1.405 (0.859, 2.301)	0.176^*^	0.598 (0.292, 1.227)	0.161
COVID-19	1.059 (0.667, 1.680)	0.808		
**Subjective social media strategies of the Shanghai Ruijin Hospital WOA**				
Providing new health knowledge	1.411 (1.150, 1.732)	0.001^*^	0.746 (0.401, 1.388)	0.355
Being an important source of health knowledge	1.341 (1.106, 1.626)	0.003^*^	1.422 (0.793, 2.549)	0.238
Professionalism and authority	1.580 (1.285, 1.944)	0.000^*^	2.354 (1.231, 4.505)	0.010^*^
Close-to-life articles	1.504 (1.224, 1.848)	0.000^*^	0.886 (0.434, 1.812)	0.741
Use of caricatures	1.533 (1.256, 1.876)	0.000^*^	1.547 (0.998, 2.398)	0.051
Use of humor	1.300 (1.064, 1.588)	0.010^*^	0.814 (0.542, 1.225)	0.324
Engagement with the public	1.032 (0.834, 1.277)	0.771		

* *p < 0.05 *.

The multivariate analysis results showed that female respondents were 4.651 times more concerned with nutrition and diet than male respondents (*p* < 0.001). Moreover, younger respondents were more likely to read nutrition and diet articles (*p* < 0.001). Regarding the knowledge acquisition approach, articles shared by a user's circle of friends, a WOA and other social media, hospitals, and television media were found to increase users' engagement with nutrition and diet articles by 2.586, 2.183, 3.194, and 4.348 times, respectively (*p* < 0.05). It was also found that users were 4.333 and 2.121 times more likely to be concerned about cancer precaution and traditional Chinese medicine articles than nutrition and diet articles (*p* < 0.001). In terms of social media strategies, professionalism and authority, were found to increase users' preference for nutrition and diet knowledge by 135.4%, respectively (*p* < 0.0001), but no effects of the use of a humorous strategy or caricatures, close-to-life articles, and engagement with the public were observed.

## Discussion

Food is an indispensable part of life, and there is an old Chinese saying that “food is the most important thing for the people.” Chronic diseases and even tumors are closely related to poor eating behaviors. Throughout the past three decades, the dietary intake of Chinese people has changed substantially (3, 30), and the current dietary structure is unreasonable. Combined with the unhealthy lifestyle factors of insufficient physical activity, smoking, and excessive drinking, the problems of overweightness, obesity, and diet-related chronic diseases are becoming increasingly more serious in China (31, 32). Obesity increases the risk of many chronic diseases, such as hypertension, cardiovascular disease, and diabetes. China spends approximately CNY 24.35 billion (USD 3.24 billion) each year to treat obesity-related diseases, accounting for 2.46% of its annual healthcare expenditures (33). Primary prevention is the most effective and affordable means by which to prevent chronic diseases, and emphasizing the quality and quantity of food may be the best measure to achieve long-term personal and social goals at each stage of the life cycle. In 2016, the Chinese government proposed the “Healthy China” strategy (9), and has issued a series of nutrition and health policies, such as the “China Nutrition Improvement Action Plan,” the “Food and Nutrition Development Strategy for China (2014–2020),” the “National nutrition plan (2017–2030),” and “Healthy China action (2019–2030),” to realize the popularization of health knowledge, an appropriate diet and national fitness, and other major actions.

Yang et al. (34) reported that Chinese adults have poor dietary knowledge, attitudes, and behaviors, while healthy dietary knowledge, attitudes, and behaviors are associated with higher self-rated health. The theory of knowledge, attitudes/beliefs, and behaviors/practices (KAB) was originally proposed to emphasize the vital role of knowledge, attitudes, and behaviors in health management (35). Sun et al. (36) found that, in China, to improve one's health, the most important measure is to increase one's own dietary knowledge. Specifically, the assessment of diet-related knowledge attitudes is of vital importance in dietary health promotion at the population level (37, 38), and whether the nutrition policies and implementation of nutrition education are of interest to the public must be determined. The results of the regression analysis conducted in the present study revealed that nutrition and diet-related health knowledge is associated with a higher influence index.

WOAs can effectively combine limited health education resources with modern science and technology and can promote people to learn about the health concepts of various diseases to effectively prevent and treat chronic diseases (39, 40). Zhang et al. (41) found that online nutrition education improved participants' knowledge of, as well as their intentions regarding, healthy dietary and lifestyle choices. A systematic review evaluated improvements in chronic disease management based on social media use, and revealed that social media, especially Facebook and blogs, provides social, spiritual, and empirical support for chronic diseases; the use of these social media platforms are thus highly likely to improve patient health (42). The present study analyzed data released by the Shanghai Ruijin Hospital WOA, and found that the nutrition and diet articles published by the WOA had the highest influence index values. This result is similar to the findings of previous research; for instance, Zhang et al. (41) found that the public likes to read and praise articles about food safety and nutrition, infectious diseases, vaccination, and healthy lifestyles.

The factors that affect people's interest in nutrition and diet knowledge were investigated in this work. A questionnaire survey was conducted, and it was found that women are more interested in nutrition and diet knowledge than men. This result is similar to the findings of previous research. For example, Lacaille et al. (43) reported that women expressed a more specific desire to eat healthily and indicated that this was of greater value to them than did men; moreover, women seemed more motivated to eat healthily. Nelson et al. (44) found that women regarded all five food motives (including health and weight concerns) to be more important than their male counterparts. Surprisingly, the multivariate logistic analysis conducted in this study showed that younger people are more concerned with nutrition and diet knowledge. Many young people have access to the internet anywhere at any time on several portable digital devices, and this has enabled more opportunities to look up information rapidly and conveniently. By managing weight and appearance *via* diet, young adults feel that they could enhance their social image, popularity, and attractiveness, and ultimately their success in finding a partner (45, 46). Despite the predominance of overweightness and obesity, the “thin and fit” ideal remains the default societal standard. Young adults note that healthy eating is ultimately within their power, and that autonomous or self-driven motivation exists.

In the present study, it was also found that social media, circles of friends, and television media affect users' concerns about nutrition and diet knowledge. Social cognitive theory considers that adults adopt the behavior of others through observation and vicariousness, and that such behavior becomes ingrained through positive outcomes. Regarding dietary behavior, this observation is multifaceted, e.g., what family and friends eat day-to-day (47). This influence is becoming increasingly notable *via* numerous social media platforms (48). A scoping review suggested the necessary use of social media platforms due to their viral nature, their reach and influence *via* peer pressure, and their popularity for health promotion (49). It was also found that the knowledge source of hospitals and the strategy of professionalism and authority affect users' concerns about nutrition and diet knowledge. Of course, healthcare professionals are trained to be literate in health matters and have information about the human body and causes of diseases. Regarding nutritionists and doctors in China's top public hospitals, their nutrition and diet knowledge represents professionalism and authority, and is favored by followers. It would be therefore worthwhile for healthcare professionals to package health information in an easily accessible manner using online resources to reach young adults and sensitize young individuals (50).

After multivariate analysis, the results showed that interest in cancer precaution and traditional Chinese medicine is connected to users' preferences for nutrition and diet knowledge, as this type of health information is inextricably linked to nutrition and diet, indicating that cancer precaution and traditional Chinese medicine knowledge related to nutrition and diet is of greater interest to the public. For example, among the 101 articles considered in this study, the influence index of the following nutrition-related cancer precaution article was ranked 11th:

*Does eating red meat cause cancer? Does eating red meat cause diabetes? Nutrition experts tell you how to eat meat*.

The high consumption of red meat (e.g., beef, pork, lamb, and goat) and processed meat (e.g., luncheon meats, frankfurters, bacon, and sausage) has been associated with the increased risk of colon, stomach, and pancreatic cancers, and with higher cancer mortality overall (51). Therefore, consuming a healthy diet aid in achieving and maintaining a healthy weight, and provides nutrients that may aid in preventing cancer. Furthermore, Miyashita et al. (52) reported that there is a need for more knowledge about healthy nutrition, which is often not satisfied, as was demonstrated in the context of a survey of Japanese breast cancer patients.

The present study also found that the type of communication used in articles is also related to user engagement. Compared with the use of a declarative sentence to convey health knowledge, articles that use humor and caricatures were found to be more likely to have a high influence index value and a higher number of “likes.” These results are in concordance with previous publications that have reported the use of humor and caricature strategies as the key features for attracting the greatest amount of user engagement, and that posts with images have higher rates of liking and sharing (53, 54).

However, nutrition science now is facing a credibility problem with the public (55–57). Although numerous discoveries and advances in the field have made great contributions to human health, nutrition science is more complex than other scientific disciplines in many aspects. According to the “2017 Tencent Rumor Governance Report,” articles related to health, health preservation, and food safety are the most numerous (58). Thus, real and scientific health information, especially that related to nutrition and diet, is particularly important for health promotion and education.

Meyrowitz proposed the theory of the “media scene,” and posited that the emergence of and changes in media will inevitably lead to changes in the social environment and human behavior (59). The mode of “new media → new scenario → new behavior” reflects the combination of media studies with social studies, the inclusion of the audience in media situations, and the change of the social structure and society *via* new media scenes. Research on health communication in China is in its infancy, and nutrition education-related research is rare. *Via* an objective analysis of real data, the present study found that nutrition and diet information is the most influential health knowledge, and then analyzed the health knowledge concerned by users and its influencing factors from the perspective of user needs. Finally, the health knowledge needed by the public was found to be consistent with the “Healthy China” health education strategy advocated by the Chinese government. *Via* social media, such as WOAs and, recently, TikTok, health education has changed, which has caused people to change their lifestyles and behaviors. Indeed, for the release of health and diet knowledge, it is suggested that an elite professional team be set up to produce reliable, authentic, rigorous, and humorous health knowledge to gain public recognition.

The results presented in this research provide hospitals or public health agencies some guidance on how they may improve health promotion and engagement with social media users. Some practical suggestions are also provided on the basis of the findings of this study. First, social media account operators can help increase information dissemination. The careful study of users' information consumption and dissemination behavior may allow operators to decide the type of information that is of interest to the public. The article content has been identified as an essential factor in determining whether WeChat users forward or share articles with friends (25). Therefore, medical institutions should fully utilize nutrition and diet knowledge to enhance diffusion. Second, the conclusions of this study could be explored in greater depth by investigating Twitter, Facebook, and/or YouTube trends in other countries, which would reflect the worldwide campaign in the domains of nutrition informatics and health dissemination. This strategy might help authorities determine what kind of information the public needs. If dissemination is efficient, the public will receive accurate information and useful prevention suggestions in a timely manner.

However, this study was characterized by some limitations. First, the methods of only a single hospital were reported and analyzed, and data from other hospitals were not collected or analyzed for comparison. However, the WOA of Shanghai Ruijin Hospital has 1,120,533 followers until June 11, 2021 and the influence of Shanghai Ruijin Hospital's WOA ranks among the top 20 hospitals in China. Thus, we consider the WOA of Shanghai Ruijin Hospital is a benchmark in the field of health promotion in China. Second, the types of social media and channels are changing rapidly, such as *via* the emergence of TikTok; due to its increasing popularity over WeChat, the construction of a TikTok group for Shanghai Ruijin Hospital has already been expedited. Third, the Shanghai Ruijin Hospital WeChat account was established in 2016, whereas the reference data considered in this study was only from 2020, when the hospital started its WOA drive. Fourth, because WeChat and mobile phones began to gain in popularity in China in recent years, these social media strategies have only been implemented for the past several years; details on longitudinal trends remain to be studied.

## Conclusions

WeChat official accounts are widely accepted media that provide health articles to users. Nutrition and health information is the most important type of health knowledge concerned by users. In this study, the factors that influence users' attention to nutrition and diet knowledge were found to be gender (female), age (young adult), contents of health knowledge (cancer precaution), and various social media strategies. Medical institutions should make full use of social media to popularize nutrition knowledge. Nutritionists or personnel engaged in nutrition education should also pay more attention to the importance of social media, not only off-line classes or outpatient consultation, to popularize the concept of nutrition and health.

## Data Availability Statement

The original contributions presented in the study are included in the article/Supplementary Material, further inquiries can be directed to the corresponding author/s.

## Ethics Statement

The studies involving human participants were reviewed and approved by Ethics and Research Committee of Ruijin Hospital. The patients/participants provided their written informed consent to participate in this study. Informed consent was obtained from all subjects involved in the study.

## Author Contributions

FZ and DB: conceptualization. DB and GL: methodology. FZ, YS, and CS: validation. DB: formal analysis and writing—original draft preparation. DL, KH, and WT: investigation. CS: data curation. GL and FZ: writing—review and editing. YS and WT: supervision. All authors have read and agreed to the published version of the manuscript.

## Funding

This research was funded by Major Key Research Project in Philosophy and Social Sciences of the Ministry of Education of the People's Republic of China (18JZD044) and also supported by Ruijin Hospital, Shanghai Jiao Tong University School of Medicine (GCQN-2019-C14).

## Conflict of Interest

The authors declare that the research was conducted in the absence of any commercial or financial relationships that could be construed as a potential conflict of interest.

## Publisher's Note

All claims expressed in this article are solely those of the authors and do not necessarily represent those of their affiliated organizations, or those of the publisher, the editors and the reviewers. Any product that may be evaluated in this article, or claim that may be made by its manufacturer, is not guaranteed or endorsed by the publisher.
