# Supplementary material for: The Influencing Factors of Nutrition and Diet Health Knowledge Dissemination Using the WeChat Official Account in Health Promotion
Source: Front Public Health. 2021 Nov 25;9:775729. doi: 10.3389/fpubh.2021.775729 (PMC8655680; doi:10.3389/fpubh.2021.775729)
Supplement: Supplementary file 1 [file Table_1.DOCX]

**Supplementary**

**Table 1** **Survey on iron deficiency among pregnant women in China**

**(Pregnant Women Investigation)**

**Survey Number: □□□**

**Instructions**

Dear readers, Thank you for your attention to Shanghai Ruijin Hospital! In order to understand your need for popular knowledge of diseases and healthy lifestyles, we will investigate your engagement-related conditions and some personal information. We declare that all the information you provided will only be used for research purposes. Please tick or fill in the relevant information according to the actual situation, and all questions are single choice by default except for special instructions.

| **A Basic demographic characteristic** | | | | | | |
| --- | --- | --- | --- | --- | --- | --- |
| A1 | Gender | □Female □Male | | | | |
| A2 | Age | □1= ≤18 □2= 18~35 □3= 35~45 □4= 45~60 □5= ＞60 | | | | |
| A3 | Education level | □1= ≤Junior College □2= Bachelor □3= Master □5= ≥PhD | | | | |
| A4 | Occupation | □1 = Agriculture, forestry, animal husbandry, fishery, e.g. farmers, hunters, herdsmen, fishermen, etc.  □2 = Production, transportation, equipment operators, e.g. drivers, operators, welders, etc.  □3 = Service, e.g. cooks, waiters, barbers, salesmen, etc.  □4 = Office staff, e.g. secretary, bank clerk, clerk, etc.  □5 = Professional and technical personnel, e.g. medical staff, teachers, lawyers, architects, IT, editors, athletes, etc.  □6 = Management personnel, e.g. government officials, factory directors, managers, administrative cadres, etc.  □7 = None  □8 = Others | | | | |
| A5 | Marriage | □1= Married □2= Single □3= Widowed □4= Divorced | | | | |
| A6 | Annual family income | □1= <30,000 yuan □2= 30,000-79,999 yuan  □3=80,000-11,999 yuan □4=12,000-19,999 yuan  □5=20,000-29,999 yuan □6= >30,000 yuan | | | | |
| **B Obtain health knowledge** | | | | | | |
| B1 | Whether you will actively acquire health knowledge? | □0=No □1= Yes | | | | |
| B2 | Where do you get health knowledge? | □1= Newspapers and books □2= Television and radio □3= Online search  □4= WeChat and other social media □5= Circle of friends □6= Off-line classes □7= Hospitals | | | | |
| B3 | How often do you look at health knowledge? | □1= Everyday □2= At least several times a month □3= Only if there's a need  □4= Never | | | | |
| B4 | When do you usually read health information? | □1= On the way to work □2= noon break □3= Work my way home  □4= At the table □5=  Before go to sleep at night □6= FT □7= Free time | | | | |
| **C Type of health knowledge** | | | | | | |
| C1 | What type of health knowledge are you most concerned about? | □1= Cancer precaution □2= Nutrition and diet □3= Chronic diseases  □4= Traditional Chinese medicine □5=  First-aid knowledge  □6= Mental health □7= Debunking health rumors □7= Digital healthcare | | | | |
| C2 | Which disease category do you want to obtain health information? | **Internal medicine system:**  □1=radiotherapy □2=rheumatism □3= Rheumatology □4=hypertension  □5=rehabilitation □6=Clinical nutrition □7=endocrinology and metabolic diseases □8= dermatology □9= neurology □10=nephrology  □11=gastroenterology □12=hematology □13=Traditional Chinese medicine □14=oncology  **Surgical system:**  □15= gynecotokology □16= ENT □17= neurosurgery  □18= Cardio-Thoracic Surgery □19= Pancreatic Surgery □20= Breast Surgery  □21= Burn and Plastic Surgery □22=ophthalmology | | | | |
| **D Gestational characteristics** | | | | | | |
|  |  | Strongly disagree | Somewhat disagree | Common | somewhat agree | Strongly agree |
| D1 | I gained new health knowledge by reading the Ruijin Hospital WOA |  |  |  |  |  |
| D2 | Ruijin Hospital WOA is an important way for me to obtain health knowledge |  |  |  |  |  |
| D3 | Articles published by Ruijin Hospital WOA are professional and authoritative |  |  |  |  |  |
| D4 | The published articles are close to life and very usefu |  |  |  |  |  |
| D5 | I wish there were more pictures and cartoons in the article |  |  |  |  |  |
| D6 | I would like to write in a more humorous style |  |  |  |  |  |
| D7 | The WOA reply message, will enhance my goodwill to the WOA |  |  |  |  |  |
